# MEMRI study - feedback of MEMS dosing history improves adherence to long-term HAART: adherence is associated with incidence of ‘blips’ in viral load

**DOI:** 10.7448/IAS.15.6.18062

**Published:** 2012-11-11

**Authors:** D White, S Aghar, S Taylor, R Cook, G Hickinbottom, A Berry, C Robertson, R Horne, V Cooper, A Lange, B Vrijens

**Affiliations:** 1 Heartlands Hospital, Hawthorn House, Birmingham, UK; 2 Center for Behavioural Medicine, UCL School of Pharmacy, London, UK; 3 Aardex Group, Vise, Belgium

## Abstract

In routine clinical care we investigated the effect on adherence to HAART of feedback to each patient of graphical plots highlighting recent errors in their dosing history, as compiled electronically using MEMS^®^. Patients established on HAART were randomised to receive either active feedback of recent dosing errors (Group A) at clinic visits, or to serve as controls (no feedback) (Group B). After 12 months the control group were un-blinded and given feedback for a further 6 months. Questionnaires were completed in the waiting room for adherence (GEEMA), Necessity/Concerns, Intrusiveness, Self-efficacy and Conscientiousness (baseline only). Those declining/excluded from using MEMS were invited to complete baseline questionnaires (Group C). Adherence was estimated from MEMS data as the average proportion of days with at least the prescribed number of doses taken between successive appointments. Drug Holidays (DH) were defined as 3 or more consecutive days without dosing. Of a cohort of 727 ~270 were approached. 180 were randomised. 147 had evaluable MEMS data (68 Group B: 79 Group A). 85 were in Group C (questionnaires only). Baseline characteristics were similar between Group A and B. Group C had a less common past history of AIDS. There was no significant difference in baseline conscientiousness between Groups A and B. Missed doses were much more likely at weekends than weekdays (OR=1.25; [1.15–1.35]). Those taking <95% of prescribed doses during the first interval between visits were defined as poor adherers, which included 69 patients (49%). Their average baseline adherence was 78%. In Group A, average adherence increased (p=0.001) to 90% after the first feedback session, while in Group B average adherence remained stable (p=0.405). In Group B, after un-blinding and start of feedback, adherence increased to 93%. Patients in Group B were significantly less likely to bring back their MEMS for reading at each appointment (p=0.031). The incidence of viral load ‘blips’ (>50 copies/ml) was significantly increased by DH (p=0.007), frequency of DH (p=0.016), length of DH (p=0.005), and missed doses during the 4 weeks before attendance (p=0.001). Feedback of electronically compiled dosing history data improves adherence to HAART treatment and appears to be an effective intervention for reducing the incidence of viral load ‘blips’. Further results including analysis of the questionnaires will be presented.

